# Research on the Microstructure and Performance Regulation of SLM 304 Steel Under Intermittent Deformation

**DOI:** 10.3390/ma19071473

**Published:** 2026-04-07

**Authors:** Huimin Tao, Linlin Ma, Bin Liao, Feng Liu, Yadong Li, Tingting Chen, Mingming Ding, Xiaomei Guo

**Affiliations:** 1 Key Laboratory of Key Technologies for Mechanical Industry Hydroelectric Power Generation Pump Turbine, Zhejiang Key Laboratory of Pumps and Turbines, Zhejiang Engineering Research Center of Advanced Water Conservancy Equipment, Zhejiang University of Water Resources and Electric Power, Hangzhou 310018, China; liaobin2022@outlook.com (B.L.); liuf@zuwe.edu.cn (F.L.); liyd@zuwe.edu.cn (Y.L.); chentt@zuwe.edu.cn (T.C.); dingliumingming@163.com (M.D.); gxm@zuwe.edu.cn (X.G.); 2 Key Laboratory of Special Equipment Safety Testing Technology of Zhejiang Province, Zhejiang Academy of Special Equipment Science, Hangzhou 310020, China; mllainin@163.com

**Keywords:** selective laser melting, stainless steel, intermittent deformation, microstructure, mechanical performance, corrosion performance

## Abstract

This paper investigates the evolution of the microstructure, mechanical performances, and corrosion resistance of selective laser melting (SLM) 304 steel under different intermittent stretching deformation step sizes, revealing the underlying evolution patterns. The results indicate that the intermittent deformation step size significantly affects the microstructure and performance of SLM 304 steel. Larger step sizes result in more complete molten pool contours, less deformation of grain and cellular structures, and a lower martensite volume fraction; smaller step sizes lead to distorted molten pools, fragmented grains, exacerbated cellular structure distortion, and increased martensite content. In terms of mechanical performances, tensile strength, nano-hardness, and elastic modulus decrease with increasing step size, while elongation increases accordingly. Corrosion resistance improves with larger step sizes, with specimens exhibiting more complete and thicker oxide films on the surface and superior pitting resistance; continuous stretching specimens exhibit the worst corrosion resistance, while the original specimens are the best. Intermittent deformation optimizes properties by regulating microstructure, providing a basis for the design of high-performance SLM 304 steel. This study provides theoretical support for the design and application of additive manufacturing stainless steel components, facilitating the engineering and industrial application of SLM technology in high-end equipment manufacturing.

## 1. Introduction

As one of the core branches of metal additive manufacturing technology, selective laser melting (SLM), with its unique technical advantages including near-rapid solidification, near-net-shape forming, and structural integration, breaks through the bottlenecks of traditional cutting and casting processes in the fabrication of complex structural components. It has been successfully applied in key fields such as aerospace, marine engineering, and high-end equipment manufacturing [1,2]. Compared with traditional manufacturing processes, SLM technology can realize the integrated forming of components with complex inner cavities, hollow structures, and gradient functions without subsequent complicated processing procedures, greatly reducing the manufacturing cost and cycle of components. Meanwhile, its near-rapid solidification characteristic can effectively refine grains and restrain elemental segregation, significantly improving the performance and service reliability of components.

304 austenitic stainless steel, as a widely used corrosion-resistant structural material, possesses excellent corrosion resistance, good plasticity, toughness, and remarkable work-hardening ability [3,4]. Moreover, with moderate raw material cost and good formability, it is one of the most widely used structural materials in the engineering application of SLM technology. However, the SLM forming process exhibits the non-equilibrium thermodynamic characteristics of “point-by-point, layer-by-layer” melting–solidification. The severe temperature gradient, extremely high cooling rate, and repeated heating–cooling cycles during forming result in a typical multi-level microstructure feature of the non-equilibrium solidification structure [5]. The unique structural characteristics, such as melt pool boundaries, columnar or cellular grains, cellular/dendritic substructures, and high dislocation density, lead to significant differences in the mechanical response, deformation mechanism, and failure behavior between SLM 304 steel and conventional forged, rolled, or cast 304 steel. For instance, the tensile strength and hardness of SLM 304 steel are remarkably higher than those of conventional forged and rolled materials, whereas its plasticity and toughness show a wider fluctuation range [6]. Meanwhile, this steel is more sensitive to loading modes, which is closely related to the dislocation configuration, substructure distribution, and residual stress state in its microstructure.

In practical engineering applications, most metal components are not subjected to a single continuous loading, but are often under discontinuous, small-amplitude, and repeated intermittent loading conditions, such as start-stop cycles of aero-engine blades, wave impact loads on offshore platform components, reciprocating loads on precision mechanical parts, etc. Stepwise intermittent deformation (step-by-step tensile-unloading cycles) can accurately reproduce the plastic deformation and micro-damage of components under intermittent loads, and builds a research bridge between monotonic tension and low-cycle fatigue, which is a key experimental method for revealing the mechanical behavior of materials under intermittent loading [7]. Compared with traditional continuous tensile deformation, obvious elastic recovery, residual strain accumulation, and cyclic hardening/softening effects exist during intermittent loading, which induces a series of complex microstructural evolutions inside the material [8]. The microstructural evolution directly affects the strength and toughness, fatigue life, plastic reserve, and structural stability of the material, and even determines the service reliability and service life of components. Therefore, an in-depth study of the microstructural evolution and macroscopic mechanical performance response of materials under stepwise intermittent deformation is of great significance for guiding the design, manufacture, and life assessment of engineering components. Previous studies have shown that unloading relaxation during intermittent loading can effectively alleviate the stress concentration inside the material. However, an overlarge loading step or excessive cycles will lead to excessive accumulation of residual strain, induce the initiation and propagation of micro-cracks, and further reduce the plasticity and fatigue performances of the material [9,10].

At present, research on SLM steel at home and abroad mainly focuses on three aspects: process parameter optimization, microstructure control, and improvement of monotonic tensile properties [11]. Process optimization often revolves around key parameters such as laser power and scanning speed. Microstructure control is achieved through heat treatment and process synergy to optimize grain morphology, refine grains, and reduce dislocation density and residual stress. The research on monotonic tensile properties focuses on macroscopic mechanical indicators and their correlation mechanism with microstructure. However, there is still a lack of systematic research on SLM steel under intermittent deformation: previous studies on intermittent deformation were mostly limited to traditional materials or monotonic loading conditions, with insufficient understanding of the complex microstructural evolution laws of SLM materials during intermittent deformation, and a lack of systematic analysis of the aging of microstructural evolution. Meanwhile, the coupling mechanism between the microstructure evolution and macroscopic mechanical properties of SLM steel after intermittent deformation has not yet been established. The above-mentioned research gaps make it difficult to accurately predict the service behavior and service life of SLM steel components under intermittent loads, and cannot provide theoretical support for the reliability design, process optimization, and life evaluation of additive manufacturing components under intermittent load conditions. This seriously restricts the engineering application of SLM technology in the field of high-end equipment components that withstand intermittent loads.

In view of this, this paper takes SLM 304 steel as the research object and carries out stepwise intermittent tensile tests with different step sizes. Combined with macroscopic mechanical performances and microstructural characterization techniques, the stress–strain response law of SLM 304 stainless steel under cyclic displacement step loading is systematically revealed. The evolution characteristics of the microstructure during intermittent deformation are analyzed in depth, and a correlation model of “intermittent deformation—microstructure evolution—macroscopic performances” is established. This study not only enriches the research on the mechanical behavior and micromechanisms of SLM-formed austenitic stainless steel under intermittent loading, but also provides a solid theoretical basis for the service behavior prediction, reliability design, process optimization, and life evaluation of additively manufactured stainless steel components under cyclic intermittent loading. It further promotes the engineering application and industrial development of SLM technology in the field of high-end equipment manufacturing.

## 2. Materials and Methods

### 2.1. Material Preparation and Deformation Process

**Figure 1:**
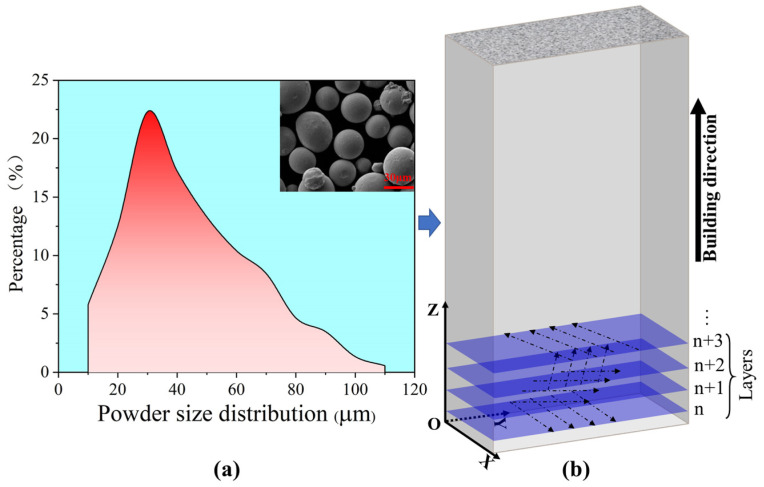
Preparation of SLM 304 steel [12]. (**a**) Stainless steel powder; (**b**) schematic diagram of selective laser melting printing.

**Figure 2:**
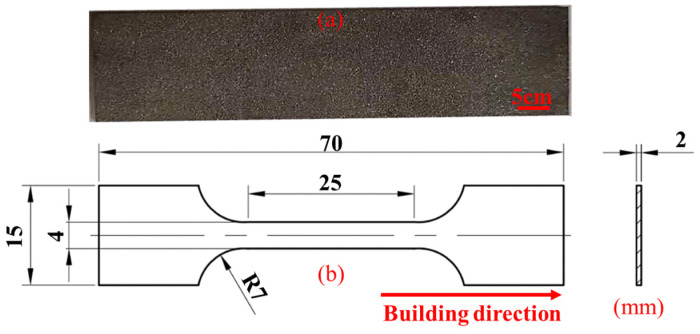
Information on tensile specimens [12]. (**a**) Printed raw materials; (**b**) two-dimensional diagram of tensile test sample.

The SLM 304 steel used in this study is shown in Figure 1. Figure 1a shows the metal powder, which was used to fabricate the material plates via an EOS M290 3D printer (GmbH Electro Optical Systems, Krailling, Germany) equipped with a 400 W Yb: YAG fiber laser. The main printing parameters are as follows: laser power 200 W, scanning speed 950 mm/s, powder layer thickness 40 μm, hatch spacing 0.07 mm, and scanning direction rotation of 67°. Figure 1b presents the schematic diagram of material preparation, and the photograph of the as-fabricated plate is displayed in Figure 2a. The printed plates were kept at 450 °C for 3 h in a furnace to relieve internal residual stress and then furnace-cooled to room temperature. The composition of the SLM 304 steel was detected through a direct reading spectrometer (Thermo Fisher Scientific, Waltham, MA, USA), and the results are listed in Table 1. The specimens were machined into tensile samples by wire electrical discharge machining (WEDM), with the dimensions shown in Figure 2b.

### 2.2. Microstructure and Performance Testing

The macrostructure and microstructure of the samples were observed using a metallurgical microscope (Leica Microsystems GmbH, Wetzlar, Germany) and a field-emission scanning electron microscope (Carl Zeiss AG, Oberkochen, Germany). The martensite content was measured with a ferrite content tester (Sungod, Suzhou, China). The oxide film on the sample surface was characterized by X-ray photoelectron spectroscopy (XPS, Thermo Fisher Scientific, Waltham, MA, USA).

Mechanical performance tests include tensile tests and nanoindentation experiments. Tensile tests were performed using an Instron 8801 universal testing machine (Instron Corporation, Norwood, MA, USA) equipped with an extensometer, in which an intermittent deformation method was adopted, i.e., multiple unloading stages were set during the stretching process, and the material was deformed under alternating loading–unloading–reloading [13]. The evolution of the microstructure and performance of SLM 304 steel was investigated by controlling the step size of each intermittent deformation. In the present study, the intermittent deformation step sizes in the tensile tests were set as uninterrupted, 1 mm, 3 mm, and 6 mm. Accordingly, the undeformed sample and the samples with different intermittent deformation step sizes were designated as As-received, Monotonic, Int-1, Int-3, and Int-6, respectively. The tensile strain rate for all samples was 5 × 10^−5^ s^−1^, and tensile tests under each condition were repeated three times to ensure the stability of the experimental results. After testing, the stress–strain curves were observed to analyze the mechanical characteristics of the specimens.

Nanoindentation tests can effectively characterize the micromechanical performance of SLM 304 steel. In these tests, a hard indenter is pressed into the sample surface under precisely controlled ultra-low load, and the load–displacement curves during indentation are recorded. Combined with theoretical models, micromechanical parameters such as nanohardness (H_n_) and elastic modulus (E_n_) are calculated. The nanohardness is calculated using Equation (1) [14,15]:

(1) $$H_{n}=\frac{P_{m}}{A_{c}}$$

(P_m_: maximum load; A_c_: actual contact projected area between the indenter and the material).

The elastic modulus is calculated using Equation (2) [14,15]:

(2) $$E_{n}=\frac{1-\nu^{2}}{\frac{1}{E_{r}}-\frac{1-\nu_{i}^{2}}{E_{i}}}$$

E_r_: Equivalent elastic modulus (including the elastic contributions of the indenter and the material); E_i_: elastic modulus of the diamond indenter (1141 GPa); ν_i_: Poisson’s ratio of the diamond indenter (0.07); ν: Poisson’s ratio of the tested material (ν = 0.3 for SLM 304 steel).

Before nanoindentation tests, all samples were electropolished in a solution containing 90% acetic acid and 10% perchloric acid to obtain a smooth surface, and the nanoindentation experiments were carried out using an Agilent G200 nanoindenter (KLA Instruments, Milpitas, CA, USA) equipped with a Berkovich diamond indenter, with a maximum load of 1.0 mN and a holding time of 5 s. At least five positions spaced 1 mm apart were tested on each specimen, and after testing, the load–displacement curves were observed, and the average nanohardness and elastic modulus were calculated.

For electrochemical corrosion tests, potentiodynamic polarization (PDP) and electrochemical impedance spectroscopy (EIS) measurements were carried out using an Ivium SXRE electrochemical workstation (Ivium Technologies B.V., Eindhoven, The Netherlands). Before the experiments, each sample was embedded in a plastic tube with epoxy resin, leaving a circular working area with a diameter of 10 mm, and a copper wire was welded to the back end. Samples were polished successively with sandpapers ranging from 400 to 2000 grit, followed by polishing with 0.5 μm and 0.1 μm diamond pastes, and ultrasonically cleaned before testing. During the measurements, the samples served as the working electrode, a platinum plate as the counter electrode, and a 232 saturated calomel electrode (SCE) as the reference electrode. All electrochemical measurements were performed in a thermostatic water bath at 25 ± 1 °C to keep the 3.5 wt.% NaCl corrosive solution at a constant temperature. For the PDP tests, the sample was immersed in the solution for approximately 10 min, and then scanned from −0.8 V_SCE_ to +0.8 V_SCE_ at a rate of 0.1 mV/s to obtain the PDP curves. The EIS tests were conducted in a frequency range from 10 mHz to 100 kHz with an alternating voltage amplitude of 10 mV. All electrochemical experiments were repeated more than 5 times to ensure reliability, and the result closest to the average value was taken as the final result. After testing, the experimental data were analyzed to evaluate the variation in corrosion resistance of different samples. Meanwhile, scanning electron microscopy was used to observe the corrosion pit distribution in different areas and to conduct a statistical analysis.

## 3. Results

### 3.1. Microstructure Characterization

**Figure 3:**
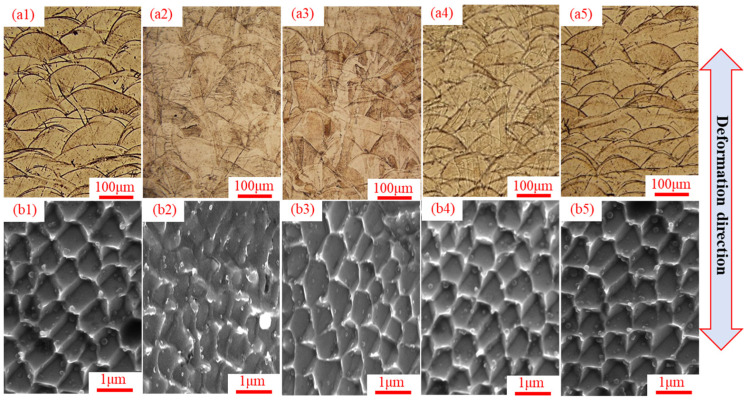
Microstructure morphology images of SLM 304 steel with different intermittent deformation step sizes. (**a1**–**a5**): Metallographic diagram; (**b1**–**b5**): Field emission scanning electron microscope image. ((**a1**,**b1**): As-received; (**a2**,**b2**): Monotonic; (**a3**,**b3**): Int-1; (**a4**,**b4**): Int-3; (**a5**,**b5**): Int-6).

Figure 3 shows the microstructure evolution of SLM 304 steel under different intermittent tensile deformation step sizes. Figure 3(a1–a5) are optical metallographic images. As shown in Figure 3(a1), the As-received sample consists of regularly arranged fish-scale molten pools with complete and clear boundaries, stacked layer by layer with varying sizes, good interlayer bonding, and no lack-of-fusion defects. In Figure 3(b1), the cellular structure exhibits regular polygons with uniform morphology, and distinct and continuous cell walls of uniform thickness. For the Monotonic sample in Figure 3(a2), the fish-scale molten pools are severely elongated, with blurred boundaries and damaged structural integrity. Meanwhile, irregular and fractured grains can be observed, and numerous slip bands appear in some regions. In Figure 3(b2), the cellular structure is elongated and flattened, with obvious deformation and partial disappearance of the cell walls, resulting in the loss of structural integrity. In the Int-1 sample (Figure 3(a3)), the fish-scale molten pools are obviously stretched with reduced boundary clarity and slight elongation. The grains are elongated, but without obvious fracture, and slip bands exist inside the grains. The cellular structure in Figure 3(b3) is slightly elongated with non-uniform morphology; the cell walls remain mostly continuous but are slightly distorted with uneven thickness. In the Int-3 sample (Figure 3(a4)), the fish-scale molten pools remain basically intact with only slight stretching, clear boundaries, and no obvious distortion, which is close to the original sample. The grains are slightly elongated with clear boundaries, and the number of intragranular slip bands is significantly reduced. The cellular structure in Figure 3(b4) shows good uniformity with a distribution similar to the original sample; the cell walls are clear and uniform in thickness, with only a few cells slightly elongated. In the Int-6 sample (Figure 3(a5)), the molten pools are complete with clear boundaries, almost identical to those of the original sample, with only very slight stretching traces in individual regions. The cellular structure in Figure 3(b5) is uniform and regular with homogeneous distribution and clear cell walls, which is basically the same as the original sample. It can be concluded that with the increase in the intermittent tensile step size of SLM 304 steel, the molten pool boundaries become more complete, grain deformation is slighter, and the cellular structure is closer to the original regular morphology. In contrast, a smaller step size leads to more severe distortion of molten pools, more obvious grain fracture, and more significant distortion of the cellular structure.

**Figure 4:**
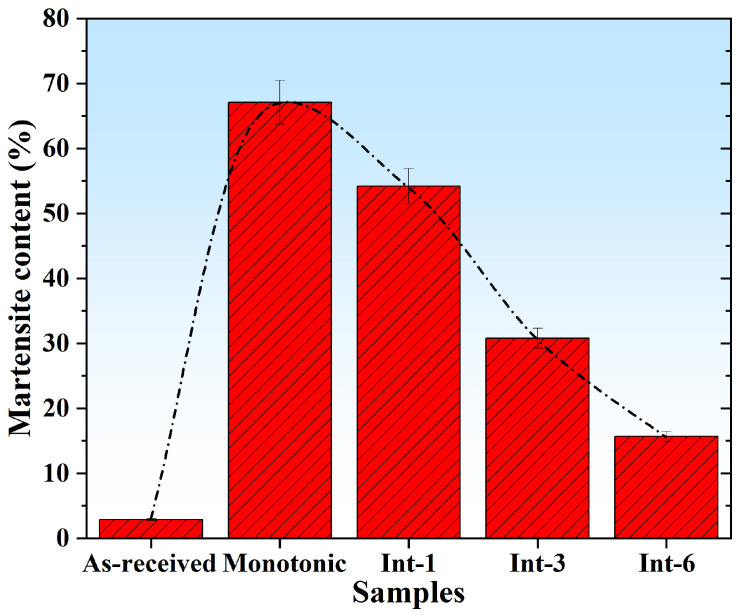
Martensitic content of SLM 304 steel with different intermittent deformation step sizes.

Figure 4 shows the phase transformation of SLM 304 steel with different intermittent deformation step sizes measured by a ferrite content tester. As can be seen from the figure, the As-received sample, without external tensile strain, exhibits an extremely low martensite volume fraction of only approximately 2.9%. The martensite content of the Monotonic sample increases significantly to about 67.1%, which is the highest among all samples. The martensite content of the Int-1 sample is about 54.2%, slightly lower than that of the Monotonic sample. The martensite content of the Int-3 sample decreases obviously to about 30.8%, while that of the Int-6 sample drops to approximately 15.7%. Overall, with the increase in the intermittent tensile step size from 1 mm to 6 mm, the martensitic transformation during tensile deformation of SLM 304 steel gradually decreases. Among all samples, the Monotonic sample shows the highest martensite content and the As-received sample the lowest.

### 3.2. Oxide Film Characteristics

**Figure 5:**
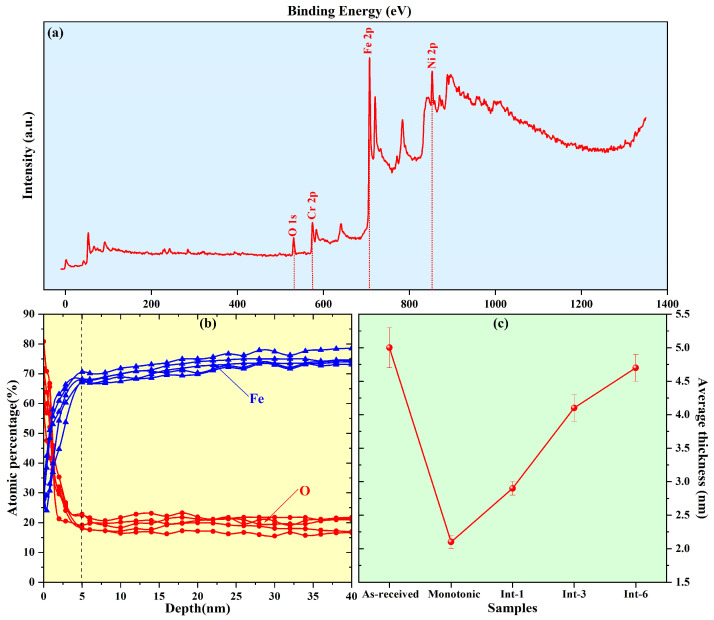
XPS study of oxide film formed on the surface of SLM 304 steel with different intermittent deformation step sizes in NaCl medium. (**a**): Composition analysis of undistorted SLM304 steel oxide film [12]; (**b**): detection of composition variation with thickness in undeformed SLM 304 steel oxide film [12]; (**c**): oxide film thickness detection of different samples.

XPS was used to analyze the composition of the surface oxide film of SLM 304 steel with different intermittent deformation step sizes after anodic polarization in NaCl solution, and the results are shown in Figure 5a. Peaks of Fe, Cr, Ni, and O were detected in the tests. The results indicate that the oxide film formed on the surface of SLM 304 steel consists of compounds related to these elements, which is consistent with the results of conventional forged 304 steel [16].

The ion etching technique coupled with XPS was adopted to further analyze the thickness of the passive film on the surface of SLM 304 steel. Since the surface film of stainless steel is mainly composed of oxides, the oxygen content in the film is significantly higher than that in the metal matrix. However, the iron content in the matrix is remarkably higher than that in the oxide film [17]. Therefore, by using high-energy ions to gradually etch from the surface to the interior of stainless steel, the thickness of the oxide film can be measured by monitoring changes in element content. The test result of the oxide film thickness for the As-received sample is presented in Figure 5b. It can be seen that the oxygen content gradually decreases with the increase in etching depth and remains basically stable at a depth of approximately 5.0 nm. For iron, an obvious turning point also appears at a depth of about 5.0 nm with increasing etching depth, and the content is higher at deeper positions. Therefore, the average thickness of the oxide film on the surface of the undeformed SLM 304 steel is about 5.0 nm. Using a similar method, the oxide film thicknesses of SLM 304 steel with different intermittent deformation step sizes were measured and counted, and the results are displayed in Figure 5c. Statistically, the oxide film thickness of the As-received sample is 5.0 nm, which is the thickest among all samples. After deformation, the oxide film thickness of the Monotonic sample decreases to approximately 2.1 nm, the thinnest of all samples. The oxide film thickness of the Int-1 sample only rebounds to about 2.9 nm. With the increase in the intermittent step size, the oxide film thickness of the Int-3 sample further increases to 4.1 nm, and that of the Int-6 sample reaches 4.7 nm, which is the closest to the As-received sample. Overall, as the intermittent tensile step size increases from 1 mm to 6 mm, the oxide film thickness on the surface of SLM 304 steel shows a gradual increasing trend. A larger intermittent tensile step size can more effectively preserve the integrity and thickness of the oxide film.

### 3.3. Mechanical Performance Testing

**Figure 6:**
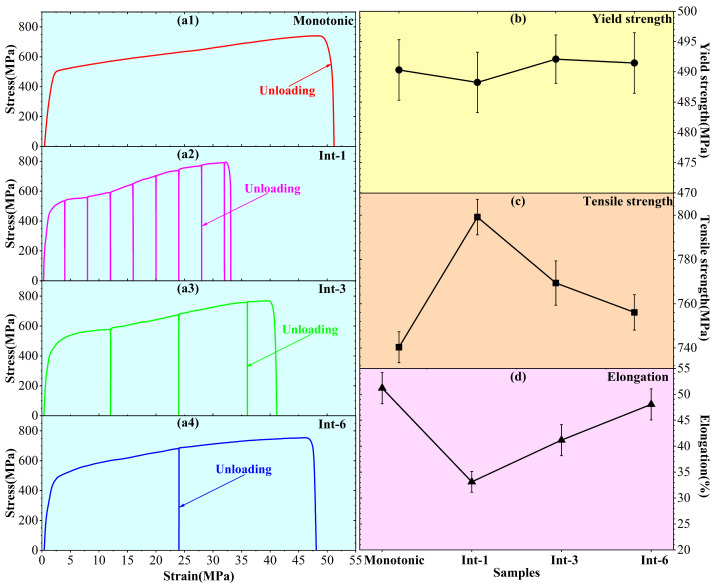
Tensile mechanical performance results of SLM 304 steel with different intermittent deformation step sizes. (**a1**–**a4**): Stress–strain curve; (**b**): Yield strength variation diagram; (**c**): Tensile strength variation diagram; (**d**): Elongation variation diagram.

Mechanical performance tests include tensile and nanoindentation tests. Figure 6 shows the tensile mechanical performances of SLM 304 steel under different intermittent deformation step sizes. Figure 6(a1–a4) present the stress–strain curves of different samples, showing distinct intermittent characteristics and significant differences in mechanical behavior. The variations in yield strength, ultimate tensile strength, and elongation of different samples are summarized in Figure 6b, Figure 6c and Figure 6d, respectively. The yield strength of all samples is similar within the error range, indicating that the intermittent tensile step size has no significant effect on the yield strength of SLM 304 steel. The ultimate tensile strength shows a decreasing trend with the increase in the intermittent step size. The specific values of the tensile test mechanical properties of different specimens are shown in Table 2. The Int-1 sample exhibits the highest ultimate tensile strength of approximately 799.15 MPa, which is notably higher than the 740.34 MPa of the Monotonic sample. The ultimate tensile strengths of the Int-3 and Int-6 samples gradually decrease to about 769.35 MPa and 756.10 MPa, respectively, approaching the level of the Monotonic sample. The variation trend of elongation is opposite to that of ultimate tensile strength, showing a clear increasing trend with the increase in the intermittent step size. The Int-1 sample has the lowest elongation of 33.12%, much lower than the 51.20% of the Monotonic sample. The elongation of the Int-3 sample increases to 41.17%, and the Int-6 sample further rises to 48.07%, which is closest to the ductility of the Monotonic sample. Therefore, intermittent deformation has little effect on the yield strength of SLM 304 steel, while the ultimate tensile strength gradually decreases and the elongation gradually increases with the increase in the intermittent deformation step size.

**Figure 7:**
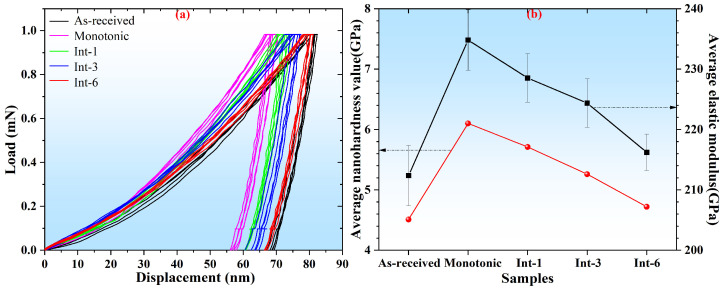
Nanoindentation test results of SLM 304 steel with different intermittent deformation step sizes. (**a**): Nanoindentation load–displacement curves; (**b**): variation diagram of nano-hardness and elastic modulus.

Figure 7 shows the nanoindentation test results of SLM 304 steel under different intermittent tensile deformations. From the load–displacement curves in Figure 7a, it can be seen that when the applied load increases to the maximum value of 1 mN and remains constant, significant differences exist in the maximum indentation depth among different samples. The curve of the Monotonic sample is obviously shifted to the left with the smallest maximum indentation depth, while the curve of the As-received sample is distinctly shifted to the right with the largest maximum indentation depth. With the increase in the deformation step size, the load–displacement curves shift obviously to the right and the maximum indentation depth increases significantly, indicating that the nanohardness of the samples gradually decreases. Figure 7b displays the statistical results of the average nanohardness and elastic modulus of different samples. The results show that the As-received sample exhibits a nanohardness of 4.51 GPa and an elastic modulus of 212.36 GPa, which are the lowest values among all samples. After tensile deformation, the Monotonic sample presents the highest nanohardness (6.10 GPa) and the largest elastic modulus (234.81 GPa). The nanohardness and elastic modulus of the Int-1 sample are 5.71 GPa and 228.52 GPa, respectively. With the increase in the intermittent step size, the nanohardness and elastic modulus of the Int-3 sample further decrease to 5.26 GPa and 224.37 GPa. When the step size continues to increase to 6 mm, the nanohardness and elastic modulus of the Int-6 sample become closer to those of the As-received sample. Overall, as the intermittent tensile step size increases from 1 mm to 6 mm, the nanohardness and elastic modulus of SLM 304 steel show a gradual decreasing trend. A larger intermittent tensile step size leads to nanohardness and elastic modulus closer to those of the original sample.

### 3.4. Corrosion Resistance Testing

**Figure 8:**
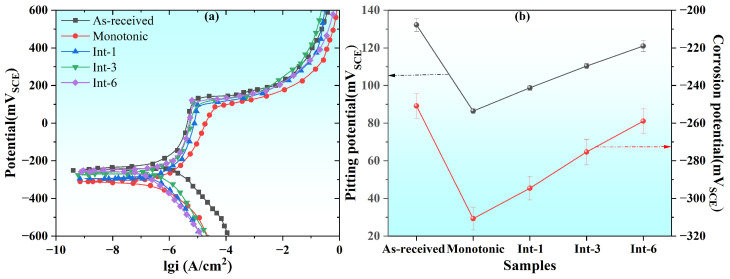
Test results of electrochemical potentiodynamic polarization of SLM 304 steel with different intermittent deformation step sizes. (**a**): Potentiodynamic polarization curve graph; (**b**): potential change graph.

Electrochemical corrosion tests, including PDP and EIS measurements, were performed on SLM 304 steel samples with different intermittent deformation step sizes. Figure 8 presents the PDP test results. The curves in Figure 8a show that the SLM 304 steel exhibits similar profiles before and after deformation, with obvious passive regions, while the key parameters of the curves vary significantly among different samples. The variations in the key parameters of different samples are shown in Figure 8b, and the specific statistical values are listed in Table 3. The results indicate that the As-received sample exhibits the best corrosion resistance, with E_p_ = 132.20 mV_SCE_ and E_c_ = −251.34 mV_SCE_, and the lowest i_p_ and i_c_ among all groups. The Monotonic sample subjected to continuous plastic deformation shows the worst corrosion resistance, with E_p_ decreased to 86.43 mV_SCE_ and E_c_ decreased to −310.71 mV_SCE_. For the intermittently tensile samples, the corrosion resistance increases gradually as the step size increases from 1 mm to 6 mm: the E_p_ and E_c_ of the Int-1 sample are 98.7 mV_SCE_ and −294.56 mV_SCE_, respectively; the Int-3 sample presents better parameters, with E_p_ and E_c_ increased to 110.32 mV_SCE_ and −275.34 mV_SCE_, respectively; the E_p_ and E_c_ of the Int-6 sample are close to those of the original sample, reaching 120.97 mV_SCE_ and −260.34 mV_SCE_, respectively. Overall, the As-received sample shows the optimal corrosion resistance, while the Monotonic sample shows the poorest. A larger intermittent tensile step size leads to PDP key parameters closer to those of the original sample and stronger corrosion resistance.

**Figure 9:**
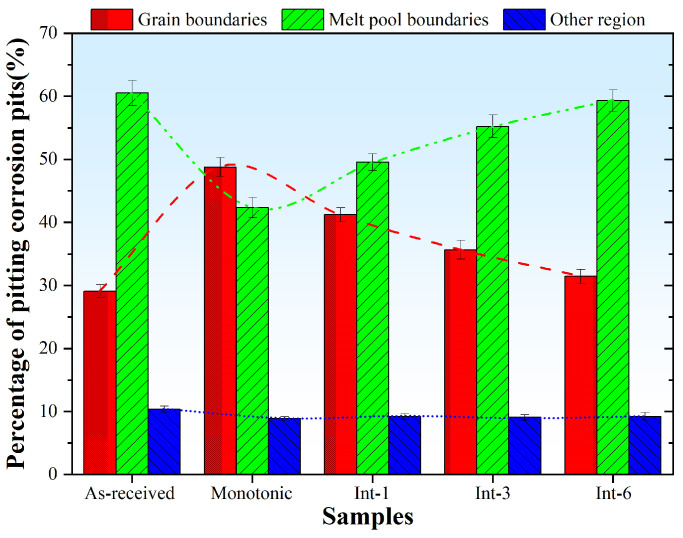
Distribution of corrosion pits after pitting corrosion of SLM 304 stainless steel with different intermittent deformation step sizes. (Red: Grain boundaries; Green: Melt pool boundaries; Blue: Other region).

Figure 9 shows the statistical results of corrosion pit distribution after pitting corrosion of SLM304 steel with different intermittent deformation step sizes. The results indicate that the corrosion pits of the As-received sample are mainly distributed at the molten pool boundaries, accounting for 60.51%, while those at the grain boundaries account for 29.11%, reflecting that the molten pool boundaries in the original SLM microstructure are more likely to act as pitting nucleation sites. For the Monotonic sample, the corrosion pit distribution changes significantly: the proportion of pits at grain boundaries increases sharply to 48.76%, while that at molten pool boundaries decreases to 42.35%, indicating that continuous plastic deformation generates new preferential nucleation sites for pitting. With the increase in the step size from 1 mm to 6 mm, the corrosion pit distribution gradually returns to the state of the original sample: the proportion of pits at grain boundaries and molten pool boundaries is 41.23% and 49.57% for the Int-1 sample, respectively; for the Int-3 sample, the proportion at grain boundaries decreases to 35.68% and that at molten pool boundaries rises to 55.24%; for the Int-6 sample, the proportion at grain boundaries further decreases to 31.45% and that at molten pool boundaries reaches 59.32%, which is closest to the distribution characteristics of the original sample. Overall, monotonic tensile deformation transforms the corrosion pit distribution from “molten pool boundary-dominated” to “grain boundary-dominated”. A larger intermittent tensile step size results in a lower proportion of corrosion pit distribution at grain boundaries and a higher proportion at molten pool boundaries, which is closer to the original sample.

**Figure 10:**
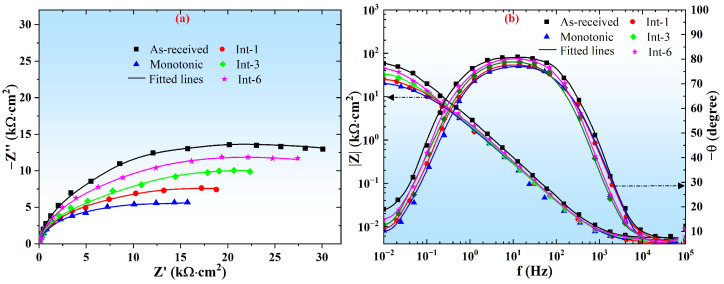
EIS test results diagram of SLM 304 steel with different intermittent deformation step sizes. (**a**): Nyquist diagram; (**b**): Bode diagram.

Figure 10 shows the EIS test results of SLM 304 steel with different intermittent deformation step sizes. Figure 10a displays the Nyquist plots, which show that all samples exhibit a semicircular capacitive impedance loop over the entire frequency range. The order of the capacitive arc radius for different samples is: As-received > Int-6 > Int-3 > Int-1 > Monotonic. Therefore, deformation degrades the corrosion resistance of the surface oxide film of SLM 304 steel, and the corrosion resistance of the oxide film increases as the intermittent deformation step size increases from 1 mm to 6 mm. Figure 10b presents the Bode plots. Within the test frequency range, the phase angle of all samples fluctuates between approximately 75° and 80°. The As-received sample shows the largest phase angle in the medium-frequency region, while the Monotonic sample shows the smallest. A larger phase angle corresponds to better capacitive behavior of the oxide film. Thus, the As-received sample exhibits the best corrosion resistance, and the Monotonic sample the worst. In addition, in the low-frequency range (0.01–1 Hz), the As-received sample has the highest impedance value, indicating the most stable surface oxide film; the Monotonic sample has the lowest impedance value, and the impedance value increases with increasing deformation step size. Therefore, the oxide film of SLM 304 steel becomes more protective with increasing deformation step size.

**Figure 11:**
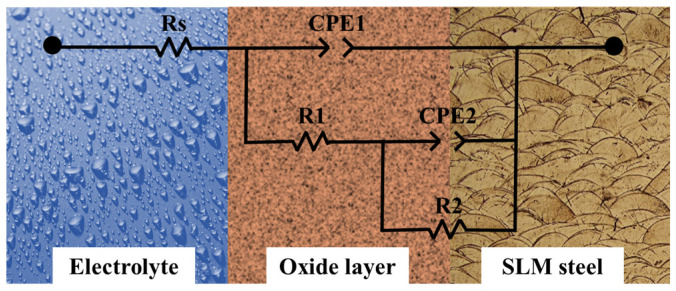
Equivalent circuit model diagram of EIS for SLM 304 steel with different intermittent deformation step sizes.

Since an oxide film forms on the surface of SLM 304 steel, the two-time-constant equivalent circuit R_s_(Q_1_R_1_(Q_2_R_2_)) shown in Figure 11 was used to fit the EIS test data. R_s_ represents the solution resistance, CPE_1_ and R_1_ represent the capacitance and resistance of the passive film, and CPE_2_ and R_2_ represent the capacitance and charge-transfer resistance of the electric double layer. The fitting parameters of the EIS results were calculated using ZView 3.1 software and are listed in Table 4. It can be seen from the table that the Rs value of each group fluctuates slightly and is not affected by the tensile deformation mode. However, the parameters R_1_, R_2_, CPE_1_, and CPE_2_, which are directly related to corrosion resistance, show significant regular changes. The As-received sample exhibits the highest R_1_ of 0.925 kΩ·cm^2^ and R_2_ of 0.876 kΩ·cm^2^, as well as the lowest CPE_1_ (3.872 × 10^−6^ F·cm^−2^) and CPE_2_ (3.215 × 10^−6^ F·cm^−2^), indicating that its passive film is intact and compact with the maximum interfacial reaction resistance. The Monotonic sample shows the worst corrosion-related impedance parameters, with R_1_ and R_2_ decreasing to 0.412 kΩ·cm^2^ and 0.234 kΩ·cm^2^, respectively, while CPE_1_ and CPE_2_ increase to 6.985 × 10^−6^ F·cm^−2^ and 7.231 × 10^−6^ F·cm^−2^, respectively, reflecting serious damage to the passive film and interface structure. With the increase in the intermittent tensile step size from 1 mm to 6 mm, the impedance parameters of the samples gradually return to those of the original sample. Among them, the R_1_ (0.898 kΩ·cm^2^) and R_2_ (0.795 kΩ·cm^2^) of the Int-6 sample are close to those of the As-received sample, and the CPE_1_ is only 4.015 × 10^−6^ F·cm^−2^. This fully indicates that intermittent tension with a large step size effectively preserves the integrity of the passive film and interface of SLM 304 steel, making its electrochemical impedance characteristics closer to the original state.

## 4. Discussion

According to the above research, the microstructure and performance of SLM 304 steel change significantly after intermittent plastic deformation. With the increase in the intermittent deformation step size, the molten pool contours in SLM 304 steel remain more intact, the degree of grain deformation decreases, and the cellular structure is closer to the original morphology. On the contrary, a smaller step size leads to more severe distortion of the molten pools, more obvious grain fracture, and more significant distortion of the cellular structure. Meanwhile, the content of transformed martensite gradually decreases with the increase in the step size. The thickness of the oxide film on the steel surface increases with the increase in the intermittent tensile step size, and the structure becomes more complete. Intermittent deformation has little effect on the yield strength of SLM 304 steel, but the ultimate tensile strength gradually decreases, and the elongation gradually increases with the increase in the deformation step size. The nanohardness and elastic modulus also show a gradual decreasing trend with the increase in the intermittent tensile step size. The pitting corrosion resistance is enhanced with the increase in the step size, and the sample under uninterrupted deformation exhibits the worst pitting corrosion resistance. It can be seen that intermittent deformation has a significant influence on the microstructure, mechanical performances, and corrosion performances of SLM 304 steel. The evolution law of the microstructure and performances of SLM 304 steel under intermittent deformation will be discussed in detail in the following section.

The effect of intermittent deformation on the microstructure of SLM 304 steel shows a clear synergistic regularity: the intermittent tensile step size is negatively correlated with the degree of microstructural deformation. That is, a larger step size results in more complete molten pool contours, slighter grain deformation, and a cellular structure closer to the original regular morphology. A smaller step size leads to more severe distortion of molten pools, more obvious grain fracture, and more significant distortion of the cellular structure. It is analyzed that the regulatory effect of intermittent tensile deformation on the molten pools, grains, and cellular structure in the metallographic structure of SLM 304 steel is essentially the synergistic influence of “stress relaxation during the intermittent pause stages” on the microstructure [18]. During uninterrupted continuous tension, stress accumulates continuously without relaxation, causing stretching and distortion of molten pools, interlayer separation, grain elongation and fracture, accumulation of slip bands, and distortion and damage of the cellular structure, eventually forming numerous microdefects and losing the overall structural integrity [19,20]. The pause stages during tension enable stress relaxation and dislocation recovery, reduce stress accumulation, and thus synergistically suppress molten pool distortion, grain deformation, and cellular structure distortion, maintaining the structural integrity of the three [21]. At a large intermittent step size (Int-6), the single strain is large, and the stress relaxation during the pause is more sufficient, which can retain the characteristics of the molten pools, grains, and cellular structure of the original SLM 304 steel to the greatest extent. During intermittent tension with a small step size (Int-1), the single strain is small, and the intermittent frequency is high, resulting in insufficient stress relaxation and obvious residual stress accumulation, leading to severe deformation of the three. The medium step size (Int-3) is between the two, with moderate stress relaxation and good microstructural stability. The original SLM 304 steel sample forms a fine cellular structure and high dislocation density due to the rapid solidification characteristic, but the martensite volume fraction is extremely low without external tensile strain. After continuous plastic deformation, the continuous accumulation of dislocations in the steel leads to increasing stress concentration, inducing a large amount of martensitic transformation and the highest martensite content. For the Int-1 sample, the short pause time and small single strain result in limited dislocation recovery and stress relaxation, and the residual strain and phase transformation driving force are still high, so the martensite content is only slightly lower than that of the uninterrupted sample. For the Int-3 sample, the single strain increases, and dislocation recovery and stress relaxation during the pause are more obvious; the subsequent phase transformation nucleation sites decrease, and the martensite content decreases significantly. For the Int-6 sample, the single strain is the largest, the stress relaxation and dynamic recovery after unloading are the most sufficient, the austenite stability is improved, the phase transformation is significantly inhibited, and the martensite content is the lowest. Therefore, with the increase in the intermittent tensile step size, the martensitic transformation in SLM 304 steel is gradually suppressed, and the volume fraction decreases gradually, resulting in the highest martensite content in the Monotonic sample and the lowest in the As-received sample.

During the uninterrupted tension of SLM 304 steel, continuous dislocation accumulation induces extensive martensitic transformation, resulting in the highest martensite content (67.1%). The high hardness and strength of the martensitic phase significantly improve the tensile strength of the material, but a large number of twins and slip bands reduce plasticity and lead to low elongation. With the increase in the intermittent step size, stress relaxation and dynamic recovery suppress martensitic transformation, leading to a gradual decrease in martensite content and weakening of the transformation-strengthening effect, thus reducing the tensile strength. Meanwhile, the reduction in martensite content lowers the proportion of brittle phases inside the material, so the elongation recovers gradually and plasticity is significantly improved [22,23]. In addition, the As-received sample obtains a basic yield strength due to its cellular structure [24]. Under uninterrupted tension, dislocations accumulate continuously and form tangles, further enhancing the dislocation-strengthening effect and slightly increasing the yield strength. During the tensile process, stress relaxation and dynamic recovery in the pause stages reduce the dislocation density and regularize the cellular structure, weakening the dislocation-strengthening effect but causing little change in the yield strength [25,26]. This is because the yield strength of SLM 304 steel is mainly determined by the high dislocation density of the original cellular structure, which is less affected by intermittent deformation. Under small-step intermittent tension (Int-1), insufficient stress relaxation results in a high dislocation density, leading to high strength and poor plasticity. Under large-step intermittent tension (Int-6), sufficient stress relaxation reduces the dislocation density, decreasing strength while improving plasticity. Meanwhile, uninterrupted tension causes molten pool distortion, grain fracture, and severe cellular structure deterioration, forming a large number of microcracks and stress concentration regions, which make the material prone to brittle fracture under high stress, showing high strength and low plasticity. As the intermittent step size increases, the molten pool contours become more complete, grain deformation is slighter, and cellular structures are more regular; internal defects decrease, and stress distribution becomes more uniform, allowing the material to maintain relatively high strength while achieving significantly improved plasticity, reflecting the synergistic optimization of strength and plasticity [27,28]. In summary, intermittent deformation realizes the gradient evolution of mechanical performances of SLM 304 steel by regulating martensitic transformation, dislocation density, and microstructural integrity. Uninterrupted tension provides high strength but poor plasticity, while large-step intermittent tension, through stress relaxation, can maintain relatively high strength and significantly improve plasticity, making the comprehensive performance closer to the original state.

After intermittent deformation, the corrosion resistance of SLM 304 stainless steel is significantly improved with increasing step size, as reflected by enhanced pitting corrosion resistance and improved integrity of the surface oxide film. The core driving force for this evolutionary trend lies in the synergistic regulation of the microstructure and passive film characteristics by the intermittent step size [29,30]. The As-received sample, supported by its cellular and molten pool structures, has no obvious deformation defects or stress concentrations, and undergoes extremely low martensitic transformation, thus forming a continuous and dense oxide film. This oxide film effectively blocks the intrusion of corrosive media, resulting in the best corrosion resistance. In contrast, under continuous plastic deformation, the Monotonic sample suffers from massive dislocation accumulation without relaxation, severe microstructure distortion, obvious molten pool distortion, grain fracture, and damaged cellular structure, accompanied by extensive martensitic transformation. Since the potential of the martensitic phase is lower than that of the austenitic matrix, micro-galvanic corrosion is prone to occur, making martensite the preferential nucleation site for pitting corrosion [31]. The distorted microstructure and abundant defects destroy the continuity of the oxide film, resulting in a thinner, looser, and more defective film layer, which greatly reduces the corrosion resistance. Meanwhile, the dominant pitting initiation site transforms from the molten pool boundary in the original sample to the grain boundary [32]. For the intermittently tensile samples, the corrosion resistance is gradually improved with increasing step size, which is essentially attributed to the regulation of the microstructure and martensitic transformation by stress relaxation and dynamic recovery during the intermittent pauses. In the Int-1 sample, stress relaxation and dislocation recovery are insufficient; the martensite content remains relatively high, the molten pool and cellular structure are obviously distorted, and the oxide film integrity is poor, so the improvement in corrosion resistance is limited. When the step size increases to 3 mm (Int-3), stress relaxation becomes more sufficient, the dislocation density and martensite content are further reduced, the molten pool contours, grain morphology, and cellular structure are gradually restored, the microdefects decrease, and the compactness and continuity of the oxide film are enhanced. As a result, the resistance to corrosive medium penetration increases, and the pitting corrosion resistance is significantly improved. In the Int-6 sample, stress relaxation is the most sufficient, the stability of austenite is improved, martensitic transformation is significantly suppressed, and the microstructure is the closest to the original state. The oxide film is intact and compact, which can effectively resist the attack of chloride ions and reduce micro-galvanic corrosion and stress concentration. Thus, the nucleation and propagation of pitting corrosion are obviously inhibited, and the corrosion resistance is greatly improved. Overall, the intermittent tensile step size controls the degree of stress relaxation and dynamic recovery, thereby regulating the microstructure integrity and martensite content of SLM 304 steel, which further determines the compactness and stability of the surface oxide film. A larger intermittent step size leads to more sufficient stress relaxation, fewer microdefects, lower martensite content, a more intact and compact oxide film, and stronger pitting corrosion resistance. Conversely, a smaller step size causes more severe microstructure distortion, higher martensite content, more serious oxide film damage, and poorer corrosion resistance. This mechanism clearly reveals the intrinsic relationship among “structure–oxide film characteristics–corrosion performance” of intermittently deformed SLM 304 steel.

In summary, different intermittent deformation conditions have a significant impact on the microstructure of SLM 304 steel, and changes in microstructure further affect its mechanical and corrosion performances. This study is of great significance: it not only enriches the service behavior and micro-mechanism of SLM-formed stainless steel under intermittent loads, filling the research gap in this field, but also provides theoretical and experimental support for the service behavior prediction, reliability design and process optimization of additive manufactured stainless steel components, promoting the engineering and industrialization development of SLM technology in the field of high-end equipment manufacturing.

## 5. Conclusions

In this paper, SLM 304 steel was subjected to intermittent tensile deformation, and the effects of different step sizes on the microstructure, mechanical performance, and corrosion resistance of the material were investigated. The main conclusions are as follows:

(1) The intermittent tensile step size has a significant effect on the microstructure of SLM 304 steel. With the increase in the step size, the molten pool contours become more complete, the grain deformation is slighter, the cellular structure is closer to the original regular morphology, and the martensite volume fraction is lower. In contrast, a smaller step size or uninterrupted tension leads to more severe molten pool distortion, grain fracture, cellular structure deterioration, and higher martensite content.
(2) Intermittent deformation has little effect on the yield strength of SLM 304 steel. The ultimate tensile strength decreases with the increase in the intermittent step size, while the elongation shows an increasing trend. The nanohardness and elastic modulus also decrease gradually with the increase in the step size. Intermittent tension with a large step size can achieve the synergistic optimization of strength and plasticity.
(3) The corrosion resistance of SLM 304 steel is improved with the increase in the intermittent tensile step size. A larger step size results in a more complete and thicker surface oxide film and stronger pitting corrosion resistance. The uninterrupted tensile sample exhibits the worst corrosion performance, while the As-received sample shows the best corrosion resistance. The microstructure integrity and martensite content are the key factors affecting the corrosion performance.

## Figures and Tables

**Table 1:** SLM 304 stainless steel composition (wt.%) [12].

Material	Si	Mn	P	S	Cr	Ni	Fe
SLM304	0.36	1.12	0.02	0.02	18.19	8.61	Bal.

**Table 2:** Mechanical performance of tensile test of SLM 304 steel with different intermittent deformation step sizes.

Samples	σ_s_/MPa	σ_b_/MPa	δ/%
Monotonic	490.31 ± 5	740.34 ± 7	51.20 ± 3
Int-1	488.25 ± 5	799.15 ± 8	33.12 ± 2
Int-3	492.10 ± 4	769.35 ± 10	41.17 ± 3
Int-6	491.46 ± 5	756.10 ± 8	48.07 ± 3

σ_s_: Yield strength; σ_b_: Tensile strength; δ: Elongation.

**Table 3:** Potentiodynamic polarization test results of SLM 304 steel with intermittent deformation step sizes.

Samples	E_p_ (mV_SCE_)	E_c_ (mV_SCE_)	I_p_ (μAcm^−2^)	I_c_ (nAcm^−2^)
As-received	132.20 ± 3.5	−250.84 ± 6.5	5.56 ± 0.6	0.51 ± 0.05
Monotonic	86.43 ± 2.2	−310.71 ± 6.7	12.38 ± 1.1	1.85 ± 0.21
Int-1	98.75 ± 2.1	−294.56 ± 6.2	9.72 ± 0.9	1.32 ± 0.17
Int-3	110.32 ± 3.4	−275.34 ± 6.4	7.45 ± 0.7	0.96 ± 0.07
Int-6	120.97 ± 3.2	−258.92 ± 6.7-	6.13 ± 0.6	0.68 ± 0.05

E_p_: Pitting potential; I_c_: Corrosion current density; E_c_: Corrosion potential; I_p_: Pitting current density.

**Table 4:** The fitting electrochemical parameter results of EIS of SLM 304 steel with different intermittent deformation step sizes.

Samples	R_s_ (Ω·cm^2^)	CPE_1_ (F·cm^−2^)	R_1_ (kΩ·cm^2^)	n_1_	CPE_2_ (F·cm^−2^)	R_2_ (kΩ·cm^2^)	n_2_
As-received	25.68	3.872 × 10^−6^	0.925	3.862 × 10^−4^	3.215 × 10^−6^	0.876	6.873 × 10^−5^
Monotonic	26.12	6.985 × 10^−6^	0.412	1.985 × 10^−4^	7.231 × 10^−6^	0.234	3.125 × 10^−5^
Int-1	25.97	5.763 × 10^−6^	0.689	2.653 × 10^−4^	5.987 × 10^−6^	0.458	4.562 × 10^−5^
Int-3	25.81	4.652 × 10^−6^	0.876	3.218 × 10^−4^	4.326 × 10^−6^	0.672	5.789 × 10^−5^
Int-6	25.73	4.015 × 10^−6^	0.898	3.647 × 10^−4^	3.458 × 10^−6^	0.795	6.542 × 10^−5^

R_s_: solution resistance; CPE_1_: constant phase element of the passive film; R_1_: resistance of the passive film; CPE_2_: constant phase element of the double layer at the film/metal interface; R_2_: charge transfer resistance.

## Data Availability

The original contributions presented in this study are included in the article. Further inquiries can be directed to the corresponding author.
